# Natural Selection on Coding and Noncoding DNA Sequences Is Associated with Virulence Genes in a Plant Pathogenic Fungus

**DOI:** 10.1093/gbe/evu192

**Published:** 2014-09-04

**Authors:** Gabriel E. Rech, José M. Sanz-Martín, Maria Anisimova, Serenella A. Sukno, Michael R. Thon

**Affiliations:** ^1^Departamento de Microbiología y Genética, Instituto Hispano-Luso de Investigaciones Agrarias (CIALE), Universidad de Salamanca, Villamayor, Spain; ^2^Computer Science Department, ETH Zürich, Universitätsstrasse 6, Zürich, Switzerland; ^3^Institute of Applied Simulation, Zürich University of Applied Sciences (ZHAW), Wädenswil, Switzerland

**Keywords:** positive selection, PAML, *Colletotrichum graminicola*, pathogenicity, arms race hypothesis

## Abstract

Natural selection leaves imprints on DNA, offering the opportunity to identify functionally important regions of the genome. Identifying the genomic regions affected by natural selection within pathogens can aid in the pursuit of effective strategies to control diseases. In this study, we analyzed genome-wide patterns of selection acting on different classes of sequences in a worldwide sample of eight strains of the model plant-pathogenic fungus *Colletotrichum graminicola*. We found evidence of selective sweeps, balancing selection, and positive selection affecting both protein-coding and noncoding DNA of pathogenicity-related sequences. Genes encoding putative effector proteins and secondary metabolite biosynthetic enzymes show evidence of positive selection acting on the coding sequence, consistent with an Arms Race model of evolution. The 5′ untranslated regions (UTRs) of genes coding for effector proteins and genes upregulated during infection show an excess of high-frequency polymorphisms likely the consequence of balancing selection and consistent with the Red Queen hypothesis of evolution acting on these putative regulatory sequences. Based on the findings of this work, we propose that even though adaptive substitutions on coding sequences are important for proteins that interact directly with the host, polymorphisms in the regulatory sequences may confer flexibility of gene expression in the virulence processes of this important plant pathogen.

## Introduction

The rapid accumulation of genome sequences and the development of powerful statistical methods, to detect signatures of adaptation, provide us an unprecedented opportunity to increase our understanding of functionally important genomic regions. Even though the main sources of adaptive characters causing phenotypic differences between organisms remain under debate (Hughes 2012), it is generally accepted that positive selection (PS) (selection in favor of advantageous mutations) plays an important role in the origin of new phenotypes (Anisimova and Liberles 2012). In fact, the evidence of selection acting on protein-coding sequences has increased enormously in the last 20 years (Fitch et al. 1991; McDonald and Kreitman 1991; Bishop et al. 2000; Bustamante et al. 2005; Aguileta et al. 2010; Rech et al. 2012). However, the high level of similarity between proteins (in number and function), from phenotypically very different organisms, and the fact that a large proportion of the nonprotein-coding DNA of eukaryotic genomes is actually functional (Kondrashov 2005; Taft et al. 2007; Raffaele and Kamoun 2012), has led to many researchers to ask whether phenotypic diversity is mainly determined by changes in protein-coding sequences or in the noncoding regulatory sequences (King and Wilson 1975; Oleksiak et al. 2002; Gasch et al. 2004; Whitehead and Crawford 2006; Wray 2007). For this reason, much attention has recently been given to understanding the function of noncoding DNA sequences, as exemplified by the human ENCODE project (ENCODE Project Consortium 2012) as well as to study the molecular evolution of these sequences (Zhen and Andolfatto 2012). Nevertheless, studies of adaptive evolution in noncoding DNA are currently restricted to model organisms including yeast (Fay and Benavides 2005; Borneman et al. 2007; Ronald and Akey 2007; Emerson et al. 2010), *Arabidopsis* (Kim et al. 2007), *Drosophila* (Andolfatto 2005; Haddrill et al. 2008), mice (Kousathanas et al. 2011), and humans (Keightley et al. 2005; Haygood et al. 2007, 2010). It is now becoming clear that natural selection acts on large portions of the noncoding genome.

In this work, we investigate patterns of selection operating on both protein-coding DNA sequences (CDSs) and noncoding intergenic and intronic sequences in a worldwide sample of eight strains of the filamentous fungus *Colletotrichum graminicola* (*Cg*). The genus *Colletotrichum* represents one of the ten most economically devastating groups of plant pathogens, causing postharvest rots and anthracnose spots and blights of aerial parts of the plant in a vast range of agronomic and horticultural crops throughout the world (Cannon et al. 2012; Dean et al. 2012). *Cg* infects maize (*Zea mays*) (LeBeau 1950; Jamil and Nicholson 1991), producing annual yield losses of more than 1 billion dollars in the United States alone (Frey et al. 2011) and having a great potential to damage agricultural ecosystems (Kamenidou et al. 2013). In addition, *Cg* is a model organism for the study of hemibiotrophic pathogens, those that begin their infection as biotrophs (keeping the host cell alive) but later switch to a necrotrophic lifestyle, killing their hosts and feeding on dead cells (Bergstrom and Nicholson 1999; O’Connell et al. 2012; Vargas et al. 2012). Interest in this haploid, clonally reproducing fungus has led researchers to develop a high-quality reference genome sequence, which is 51.6 Mb in length and is distributed among 13 chromosomes with 12,006 predicted protein-coding genes (O’Connell et al. 2012).

In order to investigate the selective pressures acting on different regions of the genome, we sequenced the genomes of seven phenotypically and geographically diverse isolates of *Cg* and jointly analyzed them together with the high-quality reference genome of *Cg* strain M1.001 (O’Connell et al. 2012). We found evidence that both protein-coding and noncoding DNA sequences of pathogenicity-related genes are under differential selective pressures compared with other genes. Moreover, the kind of selection acting at different regions of the genome is related to both gene function and the gene transcriptional regulation during maize infection. This study is the first genome-wide survey of natural selection acting on both coding and noncoding sequences in an agronomically important phytopathogenic filamentous fungus.

## Materials and Methods

### Strains, Genomic DNA Extraction, Sequencing, and Assembly

*Cg* strains were obtained from culture collections (supplementary table S1, Supplementary Material online). We selected strains representing a broad geographic distribution of populations that showed the largest phenotypic variation in terms of virulence, which was measured by the average lesion sizes observed on the highly susceptible maize inbred line Mo940 (data not shown). To ensure that the strains were virulent, each strain was inoculated onto maize plants. Once plants showed symptoms (4 dpi), we recovered conidia and grew monosporic cultures for 15–20 days on potato dextrose agar (PDA) medium (Sukno et al. 2008; Vargas et al. 2012). Genomic DNA was purified using the protocol described by (Baek and Kenerley 1998). The Internal Transcribed Spacer 1 (supplementary methods S1, Supplementary Material online) of each strain was amplified by polymerase chain reaction and sequenced to confirm their identity. Genomic DNA samples were sequenced by the Keck Center for Comparative and Functional Genomics (University of Illinois) on an Illumina HiSeq2000 system, producing over 400 million 100 bp reads with insert sizes between 400 and 500 bp (supplementary methods S2, Supplementary Material online). We assembled the *Cg* genomes by mapping reads to the reference genome of *Cg* strain M1.001 (GenBank: PRJNA37879) (O’Connell et al. 2012) and calling the consensus sequence using MAQ v0.7.1 using the default parameters (Li et al. 2008). The nucleotides in the consensus sequences were required to have a minimum mapping quality of 40 and minimum coverage of three (supplementary methods S3, Supplementary Material online). To access whole-genome pattern of polymorphisms, we performed an analysis of the empirically derived sliding-window distribution of Tajima’s *D* values (supplementary methods S4, Supplementary Material online).

### Definition of Classes of Sequences under Study

**Fig. 1.—:**
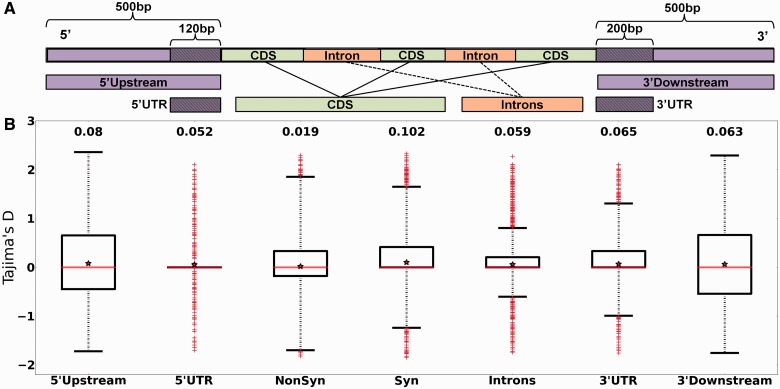
Distribution of Tajima’s *D* values for each class of sequences. (*A*) Typical eukaryotic protein-coding gene and sequence classes analyzed in the present study. (*B*) Boxplots showing the distribution of Tajima’s *D* values in each class of sequences. Values inside each box correspond to the middle 50% of the data (between the 25th [Q1] and 75th [Q3] percentiles) and the red line within the box represents the median. The ends of the vertical dotted lines (whiskers) at the top and bottom of each box indicate the maximum and minimum limits to consider outliers according to the inter quartile range (IQR = Q3-Q1). Whiskers lengths were calculated as Q3+3*IQR (upper) and Q1−3*IQR (lower). Values outside the lines (red crosses) are extreme outliers. Red stars and values at the top of the boxplots indicate mean Tajima’s *D* value for each class of sequence.

We used the genomic coordinates of the 12,006 gene models predicted in *Cg* M1.001 (O’Connell et al. 2012) to define the following classes of sequences: Coding (CDSs), introns (all introns of the gene were concatenated), 5′**-**upstream (500 bp upstream of the transcription start codon), 3′-downstream (500 bp downstream of the transcription stop codon), 5′**-**UTR (120 bp upstream the transcription start codon), and 3′-UTR (200 bp downstream the transcription stop codon) (fig. 1*A*). For all classes, we extracted genomic sequences from each consensus genome and we clustered them together to create the multiple sequence alignments. 5′**-**Upstream and 3′-downstream lengths were selected as the region expected to be enriched with regulatory elements implicated in the control of transcription and translation (Xie et al. 2005; Kousathanas et al. 2011). The length of the UTRs were defined according to the average UTR length for fungi (Mazumder et al. 2003). In all cases sequences with more than 50% ambiguously called bases (Ns) due to low read coverage or low mapping quality were discarded. We included only the intergenic regions adjacent to genes in which the start and stop codons of the reference strain’s gene (*Cg* M1.001) aligned to start and stop codons in all isolates. In addition, to preserve the identity of the noncoding sequences, we removed intergenic regions that, according the fixed lengths used in this study, overlapped with another intergenic region from a neighboring gene. Detailed protocols and scripts used in this and other analyses are available from the authors upon request.

### Site Frequency Spectrum Analysis of Different Sequence Classes

We pooled site classes across loci and used the Perl library Polymorphorama (Andolfatto 2007; Haddrill et al. 2008) to estimate Tajima’s *D* for each sequence class (introns, 5′**-**upstream, 3′-downstream, 5′-UTR, 3′-UTR and coding). To compute Tajima’s *D*, we considered only sites in the multiple sequence alignment that have unambiguously called bases from at least six strains. For coding sequences, we separately estimated Tajima’s *D* values for synonymous and nonsynonymous sites as defined by the method of (Nei and Gojobori 1986). Because we are interested in sequences showing extreme positive and extreme negative *D* values, in all cases *D* values of sequences having no polymorphic sites were set to zero. Based on the empirical distribution of Tajima’s *D* values for each class, we classified outlier values as D*: D* < 0 (*D* < 5th percentile) and D* > 0 (*D* > 95th percentile). To identify enriched functional or gene expression categories for outlier sequences, we performed Fisher’s exact tests, correcting *P* values for multiple comparisons using a false discovery rate (FDR < 0.05) separately for each class.

### Positive Selection Tests of Coding Regions

We analyzed all orthologous sets of CDSs with at least three sequences, representing a total of 11,995. Maximum-likelihood (ML) phylogenies for each orthologous set were inferred using CodonPhyML (Gil et al. 2013). Positive selection was measured by the d*N*/d*S* ratio (ω), where d*N* represents the rate of nonsynonymous substitutions per nonsynonymous site and d*S* is the rate of synonymous substitutions per synonymous site. When a coding sequence is under negative selection, nonsynonymous substitutions are constrained with respect to the neutral evolution due to their deleterious effect, and therefore ω < 1. Under neutrality, the rate of synonymous substitutions is equal to the rate of nonsynonymous substitutions (ω = 1). Alternatively, if the sequence is evolving under positive diversifying selection, d*N* > d*S* and ω > 1. We estimated ω using Markov codon models using the ML approach as implemented in the CODEML program from PAML *v4* (Yang 2007) software package. We fitted six site models of codon evolution to each sets of orthologous sequences and obtained the optimized log likelihood (lnL) values for each model. Three likelihood ratio tests (LRTs) were performed. The significance of the tests was evaluated using the LRT statistic 2*(lnL1–lnL0) = 2ΔL, which was compared with a chi-square distribution (Anisimova et al. 2001) to test whether there were statistical differences between the null (0) and the alternative (1) models. The LRTs compared the following models: M0 versus M3 to test for heterogeneity in ω among sites in a sequence, and M1a versus M2a and M7 versus M8 both to test for PS (ω > 1). We considered a sequence as evolving under PS when the LRT for the ω-heterogeneity and at least one of the LRTs for PS were significant, all with a *P* value < 0.05.

### Positive Selection Tests of Noncoding Regions

Positive selection tests of noncoding sequences were performed according to (Wong and Nielsen 2004) using the HyPhy (Kosakovsky Pond et al. 2005) batch file written by Dr Oliver Fedrigo (Haygood et al. 2007). In this analysis, the rate of nucleotide substitution in the noncoding region (d*NC*) is compared with the rate of an a priori assumed neutral rate of substitutions (d*S*) by ζ = d*NC*/d*S*. The parameter ζ represents the nucleotide substitution rate in the noncoding region, normalized by the rate of neutral substitutions (e.g., synonymous substitutions in the adjacent coding regions). Therefore, under neutrality ζ = 1, under negative selection ζ < 1, and under PS ζ > 1. We analyzed each noncoding sequence using as neutral substitution rate the pooled synonymous substitution rate of the adjacent gene as well as the upstream and downstream genes (supplementary methods S5, Supplementary Material online). We only analyzed coding and noncoding sequences present in all isolates, as previously described. The ζ values were also estimated in an ML framework, which allows us to test hypotheses concerning this parameter using LRTs. We fitted three different models to the data according to (Wong and Nielsen 2004): The neutral model (NM), the two-category model (2CM), and the three-category model (3CM), assuming no PS (ζ ≤ 1), allowing for ζ < 1 or ζ ≥ 1 and allowing for ζ < 1, ζ = 1, or ζ > 1, respectively. Two different LRTs were then performed: NM versus 2CM and NM versus 3CM for each noncoding orthologous sequence for all classes analyzed. We considered a sequence as evolving under PS when at least one LRT showed a *P* value < 0.05.

### Enrichment Analysis of Functional Categories

To investigate whether selective pressures act preferentially on specific types of sequences, we analyzed nine functional categories relevant to pathogenicity. Seven of the categories (carbohydrate-active enzymes), cytochrome P450, genus-specific effectors, secondary metabolism, secreted proteases, transcription factors, and transporters) were previously described by (O’Connell et al. 2012). Due to the extremely conservative definition of the genus-specific effector proteins as “predicted extracellular proteins without any homology to proteins outside the genus *Colletotrichum*” (O’Connell et al. 2012), we also analyzed two additional categories likely to be involved in pathogenicity: All putative secreted proteins (potentially also effectors) and putative virulence factors. We identified secreted proteins using SignalP v4.0 (Petersen et al. 2011). Putative virulence factors were annotated by performing whole-proteome BLASTp searches against the Pathogen Host Interaction Database (PhiBase v3.2) (Winnenburg et al. 2006) and against the Database of Fungal Virulence Factors (Lu et al. 2012) and we classified as putative virulence factors those genes showing at least one hit (*e* value ≤ 1e-10) in both databases. In addition, we assigned gene ontology (GO) terms to *Cg* genes using Goanna v.2 (McCarthy et al. 2006) based on sequence similarity using BLASTp. We used the UniProt and AgBase_community (Fungi) databases filtering out sequences and annotations with automatically assigned GO terms (GO evidence code: IEA). We required an *e* value ≤ 1e-5 and at least three BLAST hits with the same GO term to transfer annotations. At least one GO category was identified for 8,176 (68%) genes, and each gene was also considered to belong to all parent categories of the directly assigned GOs (Kosiol et al. 2008). We analyzed only GO terms with at least five genes. Finally, we also analyzed upregulated genes during infection at three different categories according to experimental RNA-seq data (O’Connell et al. 2012): Biotrophic/PA (significantly upregulated genes in biotrophy regarding in planta appressoria), Necrotrophic/PA (significantly upregulated genes in necrotrophy regarding in planta appressoria), and Necrotrophic/Biotrophic (significantly upregulated genes in necrotrophy regarding biotrophic phase). A detailed description of whole-genome gene annotation and gene categories analyzed in the present study is shown in supplementary table S5, Supplementary Material online. All enrichment tests were performed by creating 2×2 contingency table for the number of genes assigned or not assigned to the category and by estimating the *P* value for independence of rows and columns by the Fisher’s exact test, corrected for multiple comparisons (FDR or Bonferroni).

## Results

### Genome Sequencing, Mapping, and Whole-Genome Nucleotide Polymorphism Analysis

We analyzed whole-genome sequences of eight isolates of *Cg* from different regions of the world (table 1 and supplementary table S1, Supplementary Material online) with a variable range of virulence against maize. Mapping and assembling genomic reads from each isolate to *Cg* M1.001 reference genome resulted in high coverage consensus genome sequences for each isolate, with average read depth ranging from 24× to 132×, and coverage of at least 3× between 85% and 99% of the reference genome’s bases. The average sequence identity between isolates was 96%. The average pairwise nucleotide differences per site (Π) was 0.00301 and the genome-wide nucleotide diversity (Θ_w_) across the eight isolates was 0.00303, consistent with previous values obtained in worldwide samples of filamentous fungi (Gibbons et al. 2012).

**Table 1:** Summary Statistics and Characteristics of *Cg* Isolates

Isolate	Origin	Mapped Reads	%Used Reads	Read Depth	%Ns	SNPs	Genes
*M.1001*	Missouri, USA	Reference	—	—	9.21	—	12,006
*i318*	Nigeria	60,957,326	86.6	121×	9.83	9,170	12,004
*i113173*	Zimbabwe	58,434,572	69.8	120×	14.43	160,983	11,920
*i47511*	Michigan, USA	52,486,812	72.9	108×	13.9	141,118	11,929
*iJAB2*	Brazil	11,251,096	40.6	24×	25.24	155,561	11,900
*i13649*	Alabama, USA	46,081,744	89.7	93×	14.72	82,206	11,968
*i63127*	Germany	62,416,798	92.9	132×	19.79	115,695	11,925
*i51134*	Nagano, Japan	14,884,038	43.2	31×	19.53	139,134	11,952

Note.—Origin, region where the isolates were collected; mapped reads, the total number of effectively mapped reads from each isolate to the *Cg* M.1001 genome; %used reads, percentage of the number of sequenced reads effectively used for the assembly; read depth, the average per-base depth for each genome, taking into account only the unambiguous sites; SNPs, number of single nucleotide polymorphisms identified as compared with the *Cg* M.1001 reference genome; %Ns, for M.1001, the percentage of ambiguously called bases in the reference genome (non A, T, C, or G). For the sequenced isolates, the percentage of the genome with less than three reads coverage and therefore where SNPs were not called. Genes, number of M.1001 genes present in each isolate, considering a gene as “present” if the sequence contains more than 50% length with unambiguous bases.

In order to identify regions in the genome showing unusual patterns of polymorphism, we performed an exploratory whole-genome analysis by looking at the empirically derived sliding-window distribution of Tajima’s *D* values (supplementary analysis S1, Supplementary Material online). We found no correlation between Tajima’s *D* values and the percentage of coding sequence within sliding windows. Likewise, there was no correlation between Tajima’s *D* and the percentage of repetitive DNA (supplementary fig. S1, Supplementary Material online). However, we found a moderate negative correlation between the nucleotide diversity (Π) and the percentage of coding sequence (supplementary fig. S2, Supplementary Material online).

### Different Site Frequency Spectra between Classes of Sequences

**Fig. 2.—:**
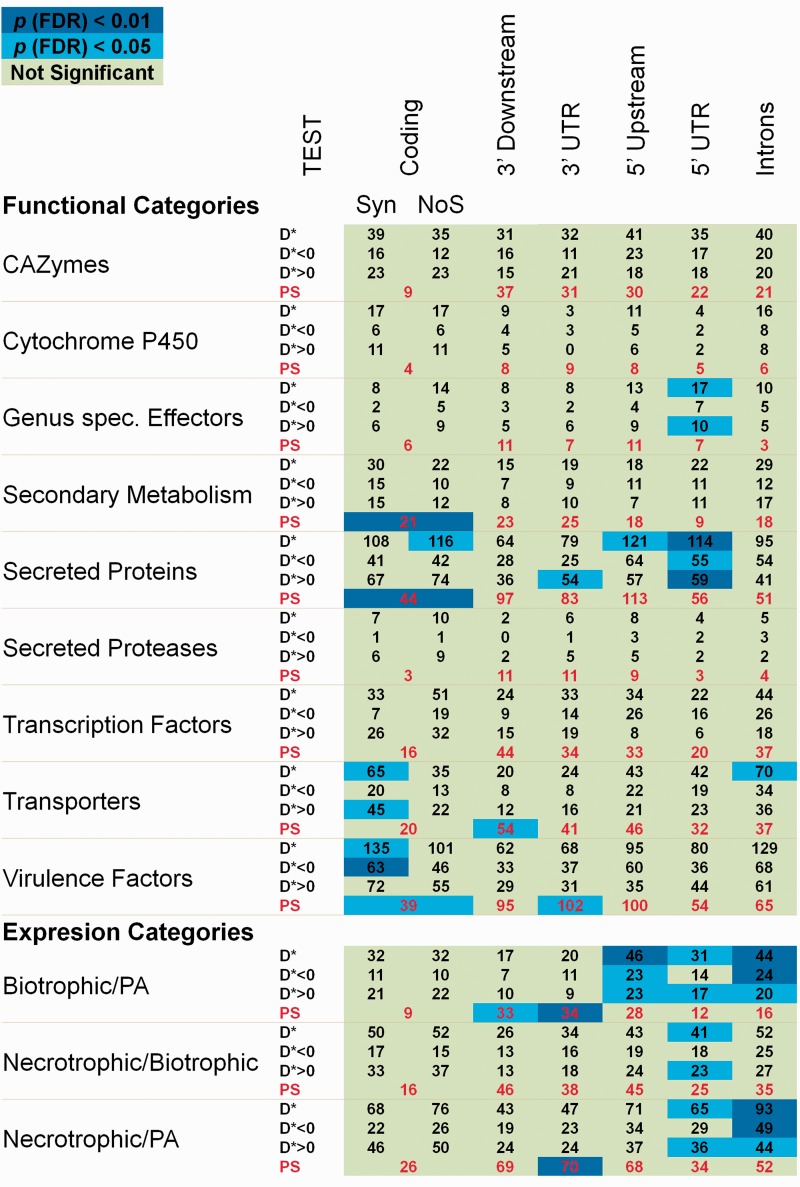
Enrichment of putative nonneutrally evolving sequences in different functional gene categories related with pathogenicity. Table values represent the number of sequences for each class and gene category. Tests: D* (*D* < 5th percentile or *D* > 95th percentile), D* < 0 (*D* < 5th percentile), D* > 0 (D > 95th percentile), and PS (sequences under PS according to LRT tests). Background colors indicate significance of the Fisher’s exact test for enrichment after correction for multiple comparisons by the FDR. See supplementary table S2, Supplementary Material online for more details.

To investigate the distribution of Tajima’s *D* values in protein-coding and noncoding DNA, we classified the entire genome into seven different classes of sequences (fig. 1*A*): introns, intergenic (5′-upstream, 3′-downstream, 5′-UTR and 3′-UTR), and coding (synonymous and nonsynonymous). Because we discarded overlapping sequences and sequences with many ambiguously called bases, we did not include all of the genes in the genome (see Materials and Methods). The mean Tajima’s *D* values were positive and very close to zero for all of the sequence classes except for synonymous sites for which the distribution was more positively skewed compared with the other classes (table 2). The distributions of polymorphism frequencies at nonsynonymous and 5′-UTR sites were the most skewed toward rare frequencies relative to synonymous polymorphisms (Wilcoxon rank-sum test versus synonymous sites: *Z* = 7.98, *P* = 1.5e-15 and *Z* = 7.28, *P* = 3.2e-13, respectively). The distribution of polymorphism frequencies in intron and 3′-UTR classes showed lower but still significant differences relative to the distribution of Tajima’s *D* values at synonymous sites (*Z* = 5.72, *P* = 1e-8 and *Z* = 3.78, *P* = 1.5e-4, respectively). However, neither 3′-downstream nor 5′-upstream regions showed differences in the synonymous distribution of Tajima’s *D* values (*Z* = 1.66, *P* = 0.09 and *Z* = 0.73, *P* = 0.46, respectively). Our results indicate that polymorphisms in introns and noncoding sequences in the immediate neighborhood of CDSs (UTRs) are more constrained on average, compared with polymorphisms at synonymous sites and at noncoding sequences further away from CDSs. By analyzing Tajima’s *D* values for each class of sequence (fig. 1*B*), we found that the 5′-UTR, 3′-UTR, and Intron sequences showed slightly positively skewed distributions of *D* values (Fisher’s skewness coefficient, *g*_1_ = 0.26, *g*_1_ = 0.15, and *g*_1_ = 0.23, respectively). We analyzed functions and transcriptional profiles of genes found in the regions with extreme Tajima’s *D* values within each sequence class by selecting outliers in the lowest and/or the highest 5% of the distribution. We separately analyzed two kinds of Tajima’s *D* outliers representing sequences with unusual patterns of polymorphisms relative to the rest of the sequences of the same class: Those showing extreme negative or extreme positive *D* values (table 2). The functional category enrichment analysis revealed that many noncoding sequences showing extreme *D* values belonged to genes related to pathogenicity (fig. 2 and supplementary tables S2 and S3, Supplementary Material online).

**Table 2:** Summary Statistics for Coding and Noncoding Sequences

	Tajima’s *D*					Positive Selection	
	Num. Seq.	D*	D* < 0 (5th Percentile)	D* > 0 (95th Percentile)	Mean Tajima's *D*	Num. Seq.	ω or ζ > 1
Synonymous	11,860	872	331 (−1.31)	541 (1.44)	0.102		
Nonsynonymous	11,860	812	309 (−1.31)	503 (1.44)	0.019 (**)	11,995	224
3′-Downstream	5,706	476	204 (−1.44)	272 (1.60)	0.063 (ns)	5,693	668
3′-UTR	9,652	537	221 (−1.31)	316 (1.44)	0.065 (*)	9,648	613
5′-Upstream	7,949	715	370 (−1.31)	345 (1.60)	0.080 (ns)	7,944	728
5′-UTR	10,733	611	329 (−1.05)	282 (1.44)	0.052 (**)	10,724	456
Introns	8,893	741	388 (−1.05)	353 (1.44)	0.059 (*)	8,742	457

Note.—Num. Seq., total number of sequences analyzed in each class. D*, number of sequences with Tajima’s *D* < 5th percentile or *D* > 95th percentile; D* < 0, number of sequences with Tajima’s *D* < 5th percentile; D* > 0, number of sequences with Tajima’s *D* > 95th percentile; mean Tajima’s *D*, symbols between brackets indicate significant differences based on a Wilcoxon rank-sum test versus synonymous (**P < 1e-10, *P < 1e-3, ns, not significant); Num. Seq. (PS), total number of sequences analyzed; ω or ζ > 1, sequences under PS in each class: Coding sequences were classified under PS when *P* < 0.05 at LRT (M0vsM3) and *P* < 0.05 at LRT (M1avsM2a) or LRT (M7vsM8). Noncoding sequences were classified under PS when any of the LRTs (NMvs2CM or NMvs3CM) showed a *P* < 0.05.

### Positive Selection in Coding and Noncoding Sequences

To investigate patterns of PS acting on both protein-coding and noncoding sequences, we applied different models of evolution aimed at comparing the nucleotide substitution rate in the region of interest with the neutral expectation. For CDSs, we fitted six Markov codon models of substitution implemented in PAML v4 (Yang 2007) to test different hypotheses regarding the estimation of the nonsynonymous to synonymous rate ratio d*N*/d*S* (also known as ω). We used LRTs to test for PS on the protein level. We performed three LRTs (M0 vs. M3, M1a vs. M2a, and M7 vs. M8) and classified coding sequences as evolving under PS when the LRT comparing M0 versus M3 showed heterogeneity of ω among sites and one of the other LRTs (M1a vs. M2a or M7 vs. M8) showed evidence for PS (all with *P* < 0.05; table 2 and supplementary table S6, Supplementary Material online). We identified 1.86% (224 out of 11,995) CDSs under PS. For most of them (203) all three LRTs were significant. The functional categories enrichment tests showed that many classes of genes previously described as evolving under PS in pathogenic fungi were also significantly enriched in our set of CDSs under PS. Such genes mainly encode for secondary metabolites, secreted proteins (putative effectors) that likely interact with host molecules and putative virulence factors (fig. 2 and supplementary table S2, Supplementary Material online). Additionally, an in-depth analysis of GO categories enriched with CDSs under PS, showed genes involved in the binding of vitamins and amino acids, in the biosynthesis of polyketides and fatty acids and genes controlling methylation (supplementary table S3, Supplementary Material online).

Noncoding sequences were classified as being under PS when any of the two LRTs (comparing models NM vs. 2CM or NM vs. 3CM) showed statistical differences supporting the model that allow for ζ > 1 (table 2 and supplementary table S6, Supplementary Material online). The 3′-downstream sequences showed the largest number of sequences putatively under PS (11.73%), whereas 5′**-**UTR were the least affected by PS (4.25%). Although we attempted to reduce the occurrence of false positives by using strict methods for base calling, it is difficult to estimate how many noncoding sequences showing PS are actually true or false positives because selection at synonymous sites, a higher mutation rate or a relaxation of selective constrains may also contribute to the signal detected by the ML test. In order to further investigate whether selection at synonymous sites were influencing our results, we analyzed the intersection of genes showing PS in the five noncoding classes. If selection at synonymous sites were biasing the detection of PS at the noncoding regions, we would expect that most noncoding sequences from the same gene show PS. We found only two genes with PS in all five classes, suggesting that selection in synonymous sites is not biasing our results (supplementary fig. S3, Supplementary Material online). An additional outcome from this analysis is the high overlap between 3′-UTR and 3′-downstream (183) and between 5′**-**UTR and 5′**-**upstream (135), which indicates that many PS signals at the more distant intergenic regions are actually coming from its contained UTR region.

## Discussion

By sequencing the genomes of seven field isolates of *Cg* and analyzing them along with the reference genome of *Cg* strain M1.001, we found that selection differentially affects coding and noncoding sequences of pathogenicity-related genes. Our first approach was to calculate Tajima’s *D* values within sliding-windows across the genome (supplementary analysis S1, Supplementary Material online). We observed a negative correlation between the nucleotide diversity and the percentage of coding sequence in the window (supplementary fig. S2, Supplementary Material online). This was expected as coding sequences are expected to be more functionally constrained than noncoding sequences. However, we also observed multiple windows with both a low percentage of coding sequence and low diversity and a high percentage of coding sequence and high diversity. In addition, we found almost no correlation between extreme Tajima’s *D* values and the percentage of coding sequence in the window (supplementary fig. S1, Supplementary Material online). These results suggest that different classes of polymorphisms in the whole window are under differential selective pressures. Although functional polymorphisms (i.e., polymorphisms that alter the gene function or its regulation) are likely to be under positive or negative selection, nonfunctional polymorphisms (i.e., polymorphisms in repetitive DNA or at synonymous sites) are more likely to be neutral. We analyzed this scenario by examining the distribution of polymorphisms in different classes of sequences.

We divided the whole genome into different classes of sequences that we expected to vary in strength and type of selection and individually estimated Tajima’s *D* for all sequences of each class. Usually, synonymous sites are considered to evolve neutrally, therefore Tajima’s *D* = 0 is expected. We obtained a positive value in our study (0.102), probably as consequence of sampling from many populations. However, such a demographic effect will equally affect all regions in the genome (Tajima 1989) and consequently differences between regions are expected to reflect the effects of selection (Bickel et al. 2013). We found reduced levels of polymorphism in UTRs, introns, and nonsynonymous sites relative to synonymous sites (table 2 and fig. 1*B*) suggesting that, on average, these sites are functionally constrained and thus under purifying selection (Andolfatto 2005). This is expected for nonsynonymous sites, because most polymorphisms will be deleterious and rapidly removed from the population. Functional constraints in UTRs and intronic sequences have been previously reported in higher eukaryotes such as *Drosophila* (Andolfatto 2005), murids (Gaffney and Keightley 2006) and *Acyrthosiphon pisum* (pea aphid) (Bickel et al. 2013). In fungi, some evidence for functional constraints in noncoding sequences were found in yeast (Gasch et al. 2004; Doniger et al. 2008) and in the filamentous pathogenic fungus *Pyrenophora* (Ellwood et al. 2012). However, even though noncoding DNA near to coding sequences are expected to be involved in the control of transcription and translation, most of them have no known function therefore selective constraints acting on these regions can sometimes be difficult to interpret (Zhen and Andolfatto 2012). Despite this, previous work found a correlation between noncoding sites under selection and the function of the adjacent gene (Gaffney and Keightley 2006). By analyzing sequences with outlying Tajima’s *D* values of each sequence class we found that many of these sequences belong to genes related to pathogenicity. For instance, 5′**-**UTR sequences with D* > 0 were significantly associated with genes encoding for secreted proteins (putative effectors) and to genes upregulated during all three stages of infection of *Cg* in maize (fig. 2 and supplementary tablesS2 and S3, Supplementary Material online). Balancing selection has been largely proposed as one of the main mechanisms responsible for the maintenance of polymorphisms in populations (Kojima 1971). However these patterns, consistent with the Red Queen model of evolution, have been mainly described for sequences encoding effector proteins in the pathogen and the corresponding resistance genes in the plant (Dodds et al. 2006; Win et al. 2007). Our results suggest that evolution of 5′**-**UTR regions, likely to be involved in the regulation of the adjacent gene, could also be driven by a Red Queen-like model, in which variability in the regulatory sequence would confer flexibility for gene expression. Organisms carrying a monomorphic pathogenicity gene, but with polymorphic regulatory sequences could show very different patterns of virulence. In contrast, 5′**-**UTRs from genes involved in the most basic functions, such as the production of cellular components or organelle organization, were underrepresented in the set with D* > 0 (supplementary table S3, Supplementary Material online), probably as a consequence of purifying selection acting on the regulatory regions from these genes.

Genes upregulated during infection also showed enrichment of intronic sequences with D* > 0 and D* < 0. There is ample evidence about the influence of introns on eukaryotic gene expression (MacKenzie and Quinn 1999; Le Hir et al. 2003; Gaffney and Keightley 2006; Albert 2011). Our results reflect the fact that some introns are expected to have a greater proportion of regulatory sequences than others, resulting in different strength and/or types of selection acting on them (Gazave et al. 2007). In addition, introns with D* > 0 were especially enriched in transporter genes (supplementary table S3, Supplementary Material online), which have been previously reported to change their transcriptional regulation due to variation in the intronic regions (Greenwood and Kelsoe 2003; Hranilovic et al. 2004). Our results show that putative regulatory noncoding sequences from many genes involved in pathogenicity are subject to different selective pressures compared with noncoding sequences from other genes.

We applied LRTs to investigate patterns of PS acting on both coding and noncoding sequences. We identified many noncoding regions in all classes showing evidence of PS (table 2 and supplementary table S6, Supplementary Material online). However, for 5′**-**upstream, 5′**-**UTR, and intronic sequences under PS, we did not find enrichment of any GO or pathogenicity-related gene category. It is possible that our approach did not allow us to identify noncoding regions under weak PS or those with just a few sites under selection. Additionally, by removing sequences with many ambiguously called bases or without well annotated start and stop codons in all of the isolates, we were actually analyzing just the most conserved sequences, because the discarded ones are likely to be the consequence of a low number of sequence reads mapping to them due to too many mismatches or genomic structural variations. 3′-Downstream and 3′-UTR sequences under PS were significantly enriched for gene categories related with pathogenicity. For instance, genes coding for transporters showed a significant enrichment of the 3′-downstream region under PS, whereas putative virulence factors and two out of the three categories of genes upregulated during infection showed enrichment of 3′-UTR under PS. These findings take relevance in light of the increasing evidence of the active contribution of 3′-UTRs in the regulation of gene expression (Thon et al. 2002; Mazumder et al. 2003; Merritt et al. 2008), suggesting that such regions could also be playing an important role in adaptations of virulence forced by the constantly changing host and environment.

At the CDS level, we found that 1.86% of the genes have evidence of PS. The PS genes are enriched with the functional categories secondary metabolism, secreted proteins, and putative virulence factors. Interestingly, putative genus-specific effectors were not enriched for CDSs under PS. Previous studies have also shown that PS genes are not always enriched with putative effectors (Stukenbrock et al. 2010; Pedersen et al. 2012) although it should be noted that the definition of effector proteins varies considerably among authors. Given the difficulty associated with the definition of effector proteins, our results could be consequence of a misclassification of effectors. This claim is partially supported by many studies that show genes of unknown function are often under PS (Li et al. 2009; Neafsey et al. 2010; Stukenbrock et al. 2010; Aguileta et al. 2012). Several authors employ a broader definition and consider that any secreted protein is a potential effector (reviewed by Doehlemann and Hemetsberger [2013]). Secreted proteins are enriched among the PS genes, which is consistent with our original expectations.

We identified 52 coding sequences under PS with no known function, of which just five were identified as genus-specific putative effectors, whereas another 17 are predicted to be secreted. Such proteins are very likely to be part of the effector repertoire and therefore are excellent candidates for functional validation (supplementary table S4, Supplementary Material online). One of these genes (*GLRG_04079*) has been functionally analyzed and is essential for pathogenicity (Vargas WA, Thon MR, Sukno SA, unpublished observations). The virulence factors category includes genes that are homologous to genes that have been shown to have some role in virulence in other fungi. However, this category may include genes involved in multiple functions. Out of 39 virulence factors identified in the present study under PS, 17 were related to the production of secondary metabolites, 8 encode transporters, 3 carbohydrate active enzymes, 2 transcription factors, 1 secreted protease, and 2 that participate in mycelium development. Secondary metabolism genes deserve special consideration in our study because *Colletotrichum* species, like most necrotrophs and hemibiotrophs, produce a diverse set of secondary metabolites, which act as antibiotics, toxins, as well as with roles in the protection from stress and pathogenicity (Keller et al. 2005; Spanu and Kämper 2010; O’Connell et al. 2012). We found that the set of genes under PS was enriched with members of this category (fig. 2), and especially with polyketide biosynthesis-related genes (supplementary table S3, Supplementary Material online). Secondary metabolism gene clusters are suspected to have undergone expansion in *Colletotrichum* species, which could shed light into the reason for finding many of them under PS. In fact, we found a new case of PS after gene duplication (Zhang et al. 1998; Emes and Yang 2008) for gene *GLRG_03511* (under PS) and its paralog *GLRG_05714*. This gene, as well other secondary metabolism-related genes under PS, may represent a source for the production of new bioactive molecules, with implications to both phytopathology and biochemistry. Overall, genes identified under PS within the coding region belong to categories previously identified in other plant pathogenic fungi as evolving adaptively (Aguileta et al. 2010, 2012; Stukenbrock et al. 2011; Gibbons et al. 2012), supporting the hypothesis that they are involved in the evolutionary arms race or in the adaptation to new environments. Some of the most interesting genes showing PS and its characteristics are listed in the supplementary table S4, Supplementary Material online, providing a valuable resource for future functional characterization.

Similar to previous studies (Aguileta et al. 2012), we did not find an enrichment of coding sequences under PS that are also upregulated during infection. A similar pattern was also found in mammals, in which coding sequences under PS showed a reduced expression level between different analyzed tissues (Kosiol et al. 2008) suggesting, according to the authors, a relationship between expression patterns and the likelihood of PS at the coding region. Alternatively, we discovered that many upregulated genes during infection show evidence for nonneutral evolution at the noncoding regions that are likely to have roles in the transcriptional regulation. King and Wilson’s hypothesis concerning the predominant role of regulatory mutations in organismal evolution (King and Wilson 1975) has lately received great support, revealing, for example, the leading role of gene expression on the local adaptations in humans (Fraser 2013). Following this line, we hypothesize that even though adaptations in the coding sequences are important for proteins expected to interact directly with the host’s molecules, and changes in regulatory sequences may drive the evolution of many other characters involved in the virulence of *Cg*. However, the role of regulatory sequence evolution remains unclear until information about genome-wide variation in gene expression, sequence polymorphisms, and phenotypic variability become available.

The present study represents the first report of selective pressure acting on both coding and noncoding DNA at the whole-genome level, for an agronomically important phytopathogenic filamentous fungus. We assessed selective pressure by aligning the sequencing reads from seven resequenced field isolates to the reference genome. Using this approach, we did not catalog structural differences among the isolates such as translocations, deletions, and insertions of DNA, some of which are also likely to be the result of selective pressure acting on the genome. In fact, such structural changes are known to affect pathogenicity-related genes in phytopathogenic fungi and have important role in adaptation. Future studies will be aimed at understanding whether differences in genome structure are correlated with differences in virulence and host range.

In this study, we found evidence that both protein-coding and noncoding DNA sequences of pathogenicity-related genes are under differential selective pressures compared with other genes. Furthermore, we found that genes coding for proteins expected to interact directly with the host’s molecules (such as effector proteins and secondary metabolites) show evidence of PS acting on the coding sequence, whereas genes upregulated during infection are enriched with UTRs and intronic DNA sequences under selective sweeps, balancing, and PS. Our findings contribute to our understanding of the evolutionary process at the molecular level and provide a valuable resource for the development of environmentally friendly strategies to control fungal diseases.

## Supplementary Material

Supplementary methods S1–S5, analysis S1, tables S1–S6, and figures S1–S3 are available at *Genome Biology and Evolution* online (http://www.gbe.oxfordjournals.org/).

Supplementary Data
