# Supplementary material for: Natural Selection on Coding and Noncoding DNA Sequences Is Associated with Virulence Genes in a Plant Pathogenic Fungus
Source: Genome Biol Evol. 2014 Sep 4;6(9):2368–79. doi: 10.1093/gbe/evu192 (PMC4202328; doi:10.1093/gbe/evu192)
Supplement: Supplementary Data [file supp_6_9_2368__index.html]

Natural selection on coding and non-coding DNA sequences is associated with virulence genes in a plant pathogenic fungus — Natural Selection on Coding and Noncoding DNA Sequences Is Associated with Virulence Genes in a Plant Pathogenic Fungus — Supplementary Data 

# Natural Selection on Coding and Noncoding DNA Sequences Is Associated with Virulence Genes in a Plant Pathogenic Fungus

## Supplementary Data

files

**Files in this Data Supplement:**

- Supplementary Data - docx file
- Supplementary Data - xlsx file
- Supplementary Data - xlsx file
- Supplementary Data - xlsx file
