# Supplementary material for: Natural Selection on Coding and Noncoding DNA Sequences Is Associated with Virulence Genes in a Plant Pathogenic Fungus
Source: Genome Biol Evol. 2014 Sep 4;6(9):2368–79. doi: 10.1093/gbe/evu192 (PMC4202328; doi:10.1093/gbe/evu192)
Supplement: Supplementary Data [file supp_evu192_Supplementary_Material.docx]

**Supplementary Material:**

**Natural selection on coding and non-coding DNA sequences is associated with virulence genes in a plant pathogenic fungus**

**Gabriel E Rech, José M Sanz-Martín, Maria Anisimova, Serenella A Sukno and Michael R Thon**

Contents

1. Supplementary Methods 2

1.1. Methods S1. Sequencing the Internal Transcribed Spacer (ITS) 1 2

1.2. Methods S2. Genomic DNA Purification and Sequencing 2

1.3. Methods S3. Sequence processing, mapping and assembly 2

1.4. Methods S4. Whole genome polymorphism analysis 3

1.5. Methods S5. Positive selection tests of non-coding regions 3

2. Supplementary Analyses 4

2.1. Analysis S1. Whole-genome nucleotide polymorphism analysis 4

3. Supplementary Tables 6

Table S1. Additional information for isolates used in the present study. 6

Table S2. Enrichment tests of Tajima’s D outliers (A) and sequences under Positive Selection (B) for gene categories associated with pathogenicity. 7

Table S4. Interesting genes under positive selection in the coding sequence. 9

4. Supplementary Figures 11

Fig. S3. Venn diagram showing gene intersections between different classes of non-coding sequences under positive selection. 12

5. References 13

# 1. Supplementary Methods

## 1.1. Methods S1. Sequencing the Internal Transcribed Spacer (ITS) 1

Prior to genome sequencing, we verified the identity of the isolates by sequencing the ribosomal RNA gene Internal Transcribed Spacer 1 (ITS1). Primers ITS5 (*GGAAGTAAAAGTCGTAACAAGG*) and ITS4 (*TCCTCCGCTTATTGATATGC*) were used to amplify ITS region 1 from all isolates. *gDNA* was used as template for PCR with the following conditions: 94°C (2’), 35 cycles of 94°C (30’’), 55°C (30’’) and 72°C (45’’) and a final extension step of 72°C (5’). We performed all PCR reactions with Biotools DNA polymerase. PCR amplifications were visualized by agarose (0.8%) gel electrophoresis at 90V, confirming the amplicon size. The amplified ITS fragments were purified using NucleoSpin Gel and PCR Clean up (Macherey Nagel) kits and sequenced at the Sequencing Service of the University of Salamanca.

## 1.2. Methods S2. Genomic DNA Purification and Sequencing

We incubated mycelia from monosporic cultures with orbital shaking in Fries’ medium (Vaillancourt and Hanau 1992) for four days, at 25°C and 150 rpm with continuous illumination and then we used the Maxi DNA extraction protocol adapted from (Baek and Kenerley 1998) to extract genomic DNA (gDNA). gDNA (1µg/100µL) was sent to the Keck Center for Comparative and Functional Genomics (University of Illinois) where shotgun DNAseq libraries were prepared with Illumina's TruSeq DNAseq Sample Prep kit. Libraries were then pooled, quantified by qPCR and sequenced on one lane for 100 cycles from each end of the fragments on an Illumina HiSeq2000 system.

## 1.3. Methods S3. Sequence processing, mapping and assembly

We assessed the quality of the reads using the FastQC software. Most isolates passed all quality controls. Two isolates (i51134 and iJAB2) failed in the “per base quality score”, so we re-sequenced these libraries, but FastQC still failed for these isolates after re-sequencing. Warn/Fail results in FastQC are common for very small genomes according to the manufacturer. In fact, the total number of sequenced reads for these two isolates was much lower than for the others. We presume that FastQC results as well as the lower number of sequenced reads could be due to problems with gDNA quality for isolates i51134 and iJAB2. Anyway, we were extremely strict when mapped reads to the *Cg* M1.001 reference genome, to call the consensus sequence and to identify SNPs, which was reflected at both the lower read depth and the greater percentage of ambiguously called based for isolates i51134 and iJAB2. Read mapping was performed using MAQ *v0.7.1* (Li et al. 2008), allowing a maximum of two mismatches per read in the first 24 bp (default parameters) for the mapping and nucleotides in consensus sequences were required to have a minimum mapping quality of 40, a minimum neighboring quality of 20 a minimum read depth of 3X and a minimum of three reads calling the same nucleotide. All regions where these requirements were not met were masked with “Ns” in all genomes and were not used for the SNP calling. Due to the highly strict filtering of reads, many of them were discarded for the analysis (between 7% of isolate i63127 and 60% for isolate iJAB2, see Table 1 in main text).

## 1.4. Methods S4. Whole genome polymorphism analysis

We concatenated all of the nuclear chromosomes together and analyzed them using Variscan v2.0.3 (Hutter et al. 2006). We calculated Tajima’s D values in 5 kb windows, using both, 500 bp step size (similar to that used by Gibbons et al., 2012), comprising a total of 98,624 windows and 5 kb step size (non-overlapping windows), comprising 9,862 windows (for practical reason, most results referred to the 500 bp step size strategy, but there was no significant difference between the two approaches). All sites with more than three ambiguities were excluded from the analysis. We classified outlier windows according to the empirical distribution of Tajima’s D values as Extreme Windows (EWs) when D<5th percentile (-1.33) or D>95th percentile (1.51). Additionally, we calculated the percentage of coding sequences in each window based on the Cg M.1001 genome annotation, and the percentage of repetitive DNA in each window according to the annotation of transposable elements (O’Connell et al. 2012) in order to perform a point-biserial correlation test (rpbi) between the membership to a EW (1) or not (0) and the percentage of coding or repetitive DNA in the window. Genes were considered as belonging to an EW when at least 50% of the genes fit in the EW. Genome-wide diversity values (ϴw and Π) were also calculated using Variscan v2.0.3 (Hutter et al. 2006).

## 1.5. Methods S5. Positive selection tests of non-coding regions

For non-coding sequences, we applied combined DNA and codon models developed by (Wong and Nielsen 2004) to compare the rate of nucleotide substitutions in the non-coding regions with the rate of synonymous substitutions in the nearby coding regions, measured by parameter ζ. However, instead of just using the synonymous substitution rate for the nearest coding sequence, we also used the synonymous substitutions rate of the pooled CDSs from the upstream and downstream genes. With this approach we expected to avoid, at least partially, the bias in synonymous substitutions rate with respect to individual gene history (Haygood et al. 2007).

# 2. Supplementary Analyses

### 2.1. Analysis S1. Whole-genome nucleotide polymorphism analysis

We examined whole-genome polymorphisms by analyzing the empirically derived sliding-window distribution of Tajima's D values in order to identify regions in the genome showing unusual patterns of nucleotide polymorphism (**Supplementary Figure S1, Supplementary Material online**). Tajima’s D reflects the difference between two estimators of the population mutation rate: the Watterson’s estimator of nucleotide diversity per site (ϴw) and the average pairwise nucleotide differences per site (Π). ϴw is influenced by the number of segregating sites (it assigns the same importance to all polymorphisms), while Π is more affected by frequent polymorphisms. Under neutrality, the average of ϴw and Π are expected to be equal, so Tajima’s D = 0. Significant deviations from zero (e.g. an excess of low or high frequency polymorphisms) represent a signal of non-neutrally evolving sequence (e.g. D > 0: balancing selection or D < 0: positive or purifying selection).

A potential problem with neutrality tests is that they can be difficult to interpret because of the confounding effects of demographic processes and/or sequencing errors (Achaz 2008) (e.g. D > 0 as a consequence of population subdivision, D<0 due to population expansion or D<0 due to large ϴw values derived from randomly called SNPs from sequencing errors). However, since such events are expected to affect all regions in the genome equally (Bickel et al. 2013), looking for unusual values of the empirical distribution of Tajima’s D values is a valid approach to identify genomic regions under selection (Przeworski et al. 2000; Carlson et al. 2005; Biswas and Akey 2006; Kelley et al. 2006). We classified windows with outlier Tajima’s D values as Extreme Windows (EWs) if Tajima’s D values were greater than the 95th percentile and lower than the 5th percentile of their genomic distribution. We identified 2,040 genes present in EWs (see Materials and Methods for a description on how genes were selected). In addition, we divided EWs into positive EWs (D>0) and negative EWs (D<0). Negative EWs are expected to contain the most selectively constrained genes since they represent windows with excess of low frequencies polymorphisms, likely to be a consequence of positive or negative selection, whereas positive EWs are expected to contain sequences with an excess of intermediate frequency polymorphisms mainly as a consequence of balancing selection or relaxation of selective constraints. A gene ontology (GO) enrichment test did not reveal an enrichment of functional categories in either set of genes after correction for multiple comparisons. However, we found an extremely weak positive correlation between EWs and the percentage of coding sequences in the window (rpbi = 0.096, p<0.0001) and a negative but stronger correlation with the percentage of repetitive DNA in the window (rpbi = -0.118, p<0.0001) (**Supplementary Figure S1, Supplementary Material online**). In addition, we found a moderate negative correlation (r = -0.53, p<0.0001) between the average pairwise nucleotide differences per site (Π) and the percentage of coding sequence in the windows (**Supplementary Figure S2, Supplementary Material online**).

A negative correlation with the percentage of repetitive DNA is expected since most polymorphisms in repetitive DNA are likely to evolve neutrally and not to be under selection (Gaffney and Keightley 2006). However, the weak correlation of most extreme windows with the percentage of coding sequences and the lack of enrichment of GO functional categories may be suggesting that different class of polymorphisms at the whole window are under differential selective pressures. While functional polymorphisms (i.e. polymorphisms that alter the gene function or its regulation) are likely to be under positive or negative selection, non-functional polymorphisms (i.e. polymorphisms at repetitive DNA or at synonymous sites) are more likely to be neutral.

# 3. Supplementary Tables

**Table S3.** Gene Ontology enrichment analysis. Only gene sets showing significant enrichment of GO terms are presented. (File: ***Supplementary Table S3.xlsx***)

**Table S5.** Whole genome gene annotation and gene categories under study. (File: ***Supplementary Table S5.xlsx***)

**Table S6.** Maximum Likelihood analysis of positive selection (PS) in coding and non-coding sequences. (File: ***Supplementary Table S6.xlsx***)

| **Table S1**. **Additional information for isolates used in the present study.** | | | |
| --- | --- | --- | --- |
| **Isolate** | **Other name/code** | **Origen** | **Source** |
| **M1.001** | CgM2 /  CBS-130836 | Missouri | Dr. Lisa Vaillancourt, University of Kentucky |
| **i318** | LARS 318 | Nigeria | Warwick (HRI) Genetic Resources Unit, UK |
| **i113173** | CBS113173 /  IMI 84302 | Zimbabwe | Common Access to Biological Resources and Information (CABRI) |
| **i47511** | NRRL47511 | Michigan | USDA-ARS Culture Collection (NRRL), USA |
| **iJAB2** | **-** | Brazil | Warwick (HRI) Genetics Resources Unit, UK |
| **i13649** | NRRL13649 - ATCC 34167 | Alabama | USDA-ARS Culture Collection (NRRL), USA |
| **i63127** | 63127 (DSMZ) | Germany | Deutsche Sammlung von Mikroorganismen und Zellkulturen |
| **i51134** | MAFF511343 | Nagano | National Institute of Agrobiological Sciences, Japan |

| **Table S2. Enrichment tests of Tajima’s D outliers (A) and sequences under Positive Selection (B) for gene categories associated with pathogenicity.** | | | | | | | | | | | | | | | |
| --- | --- | --- | --- | --- | --- | --- | --- | --- | --- | --- | --- | --- | --- | --- | --- |
| **A) Tajima's D outliers** | | | | | | | | | | | | | | | |
| **Functional Categories** | **Tajima D outliers** | | **Syn** | | **NonSyn** | | **3' Downstream** | **3' UTR** | | **5' Upstream** | | **5'UTR** | | **Introns** | |
| CAZymes | D*: | | 39/487 (4.65E-01) | | 35/487 (4.58E-01) | | 31/255 (1.81E-01) | 32/412 (1.54E-01) | | 41/398 (5.83E-01) | | 35/446 (9.88E-02) | | 40/379 (1.32E-01) | |
|  | D<0: | | 16/487 (5.15E-01) | | 12/487 (7.98E-01) | | 16/255 (1.77E-01) | 11/412 (7.74E-01) | | 23/398 (2.97E-01) | | 17/446 (4.71E-01) | | 20/379 (3.28E-01) | |
|  | D>0: | | 23/487 (5.20E-01) | | 23/487 (4.90E-01) | | 15/255 (7.58E-01) | 21/412 (1.32E-01) | | 18/398 (8.14E-01) | | 18/446 (1.23E-01) | | 20/379 (2.61E-01) | |
| Cytochrome P450 | D*: | | 17/142 (8.75E-02) | | 17/142 (5.11E-02) | | 9/71 (4.03E-01) | 3/116 (9.60E-01) | | 11/83 (5.55E-01) | | 4/121 (9.36E-01) | | 16/130 (1.32E-01) | |
|  | D<0: | | 6/142 (5.15E-01) | | 6/142 (3.74E-01) | | 4/71 (5.58E-01) | 3/116 (8.97E-01) | | 5/83 (5.15E-01) | | 2/121 (8.90E-01) | | 8/130 (3.28E-01) | |
|  | D>0: | | 11/142 (2.70E-01) | | 11/142 (9.87E-02) | | 5/71 (7.58E-01) | 0/116 (1.00E+00) | | 6/83 (4.52E-01) | | 2/121 (9.35E-01) | | 8/130 (2.61E-01) | |
| Genus-specific Effectors | D*: | | 8/171 (9.41E-01) | | 14/171 (4.58E-01) | | 8/97 (8.63E-01) | 8/139 (6.88E-01) | | 13/128 (6.57E-01) | | 17/148 (1.99E-02) | | 10/100 (3.61E-01) | |
|  | D<0: | | 2/171 (9.95E-01) | | 5/171 (6.92E-01) | | 3/97 (9.90E-01) | 2/139 (9.44E-01) | | 4/128 (8.54E-01) | | 7/148 (4.71E-01) | | 5/100 (4.99E-01) | |
|  | D>0: | | 6/171 (7.98E-01) | | 9/171 (4.90E-01) | | 5/97 (7.58E-01) | 6/139 (4.56E-01) | | 9/128 (4.52E-01) | | 10/148 (2.54E-02) | | 5/100 (5.47E-01) | |
| Secondary Metabolism | D*: | | 30/292 (8.75E-02) | | 22/292 (4.58E-01) | | 15/128 (4.03E-01) | 19/243 (1.88E-01) | | 18/169 (5.83E-01) | | 22/269 (1.24E-01) | | 29/241 (8.35E-02) | |
|  | D<0: | | 15/292 (7.70E-02) | | 10/292 (4.17E-01) | | 7/128 (5.20E-01) | 9/243 (4.77E-01) | | 11/169 (2.97E-01) | | 11/269 (4.71E-01) | | 12/241 (4.64E-01) | |
|  | D>0: | | 15/292 (5.20E-01) | | 12/292 (7.54E-01) | | 8/128 (7.58E-01) | 10/243 (4.56E-01) | | 7/169 (8.14E-01) | | 11/269 (1.47E-01) | | 17/241 (6.93E-02) | |
| Secreted | D*: | | 108/1334 (2.65E-01) | | 116/1334 (3.08E-02) | | 64/708 (5.78E-01) | 79/1131 (1.54E-01) | | 121/1081 (4.21E-02) | | 114/1226 (3.95E-07) | | 95/1024 (2.03E-01) | |
|  | D<0: | | 41/1334 (5.15E-01) | | 42/1334 (3.74E-01) | | 28/708 (5.60E-01) | 25/1131 (9.10E-01) | | 64/1081 (2.05E-01) | | 55/1226 (2.03E-02) | | 54/1024 (2.57E-01) | |
|  | D>0: | | 67/1334 (4.81E-01) | | 74/1334 (8.08E-02) | | 36/708 (7.58E-01) | 54/1131 (2.33E-02) | | 57/1081 (4.52E-01) | | 59/1226 (2.13E-05) | | 41/1024 (6.45E-01) | |
| Secreted Protease | D*: | | 7/110 (9.10E-01) | | 10/110 (4.58E-01) | | 2/56 (9.54E-01) | 6/90 (6.88E-01) | | 8/85 (7.52E-01) | | 4/101 (9.36E-01) | | 5/99 (9.24E-01) | |
|  | D<0: | | 1/110 (9.95E-01) | | 1/110 (9.46E-01) | | 0/56 (1.00E+00) | 1/90 (9.44E-01) | | 3/85 (8.54E-01) | | 2/101 (8.90E-01) | | 3/99 (8.13E-01) | |
|  | D>0: | | 6/110 (5.20E-01) | | 9/110 (9.87E-02) | | 2/56 (9.20E-01) | 5/90 (3.96E-01) | | 5/85 (6.95E-01) | | 2/101 (9.35E-01) | | 2/99 (9.09E-01) | |
| Transcription Factor | D*: | | 33/550 (9.41E-01) | | 51/550 (5.11E-02) | | 24/308 (8.63E-01) | 33/460 (1.88E-01) | | 34/399 (7.79E-01) | | 22/511 (9.36E-01) | | 44/465 (2.63E-01) | |
|  | D<0: | | 7/550 (9.95E-01) | | 19/550 (3.74E-01) | | 9/308 (9.90E-01) | 14/460 (5.09E-01) | | 26/399 (2.28E-01) | | 16/511 (8.11E-01) | | 26/465 (2.57E-01) | |
|  | D>0: | | 26/550 (5.20E-01) | | 32/550 (9.87E-02) | | 15/308 (7.58E-01) | 19/460 (3.96E-01) | | 8/399 (9.97E-01) | | 6/511 (9.35E-01) | | 18/465 (6.52E-01) | |
| Transporter | D*: | | 65/662 (4.30E-02) | | 35/662 (9.61E-01) | | 20/313 (9.54E-01) | 24/555 (9.60E-01) | | 43/510 (7.79E-01) | | 42/622 (2.51E-01) | | 70/623 (4.83E-02) | |
|  | D<0: | | 20/662 (5.84E-01) | | 13/662 (9.46E-01) | | 8/313 (9.90E-01) | 8/555 (9.44E-01) | | 22/510 (8.54E-01) | | 19/622 (8.11E-01) | | 34/623 (2.57E-01) | |
|  | D>0: | | 45/662 (4.16E-02) | | 22/662 (9.08E-01) | | 12/313 (9.20E-01) | 16/555 (9.49E-01) | | 21/510 (8.14E-01) | | 23/622 (1.23E-01) | | 36/623 (6.93E-02) | |
| Virulence Factor | D*: | | 135/1438 (1.17E-02) | | 101/1438 (4.58E-01) | | 62/756 (8.63E-01) | 68/1227 (6.88E-01) | | 95/1127 (7.79E-01) | | 80/1340 (5.07E-01) | | 129/1291 (5.6E-02) | |
|  | D<0: | | 63/1438 (1.45E-03) | | 46/1438 (3.74E-01) | | 33/756 (5.20E-01) | 37/1227 (4.21E-01) | | 60/1127 (2.97E-01) | | 36/1340 (8.90E-01) | | 68/1291 (2.57E-01) | |
|  | D>0: | | 72/1438 (4.81E-01) | | 55/1438 (9.08E-01) | | 29/756 (9.20E-01) | 31/1227 (1.00E+00) | | 35/1127 (9.97E-01) | | 44/1340 (1.23E-01) | | 61/1291 (2.37E-01) | |
| **Expresion Categories** | Tajima D outliers | | **Syn** | | **NonSyn** | | **3' Downstream** | **3' UTR** | | **5' Upstream** | | **5'UTR** | | **Introns** | |
| Biotrophic/  PA | D*: | | 32/378 (3.38E-01) | | 32/378 (1.24E-01) | | 17/192 (5.75E-01) | 20/320 (3.71E-01) | | 46/305 (8.92E-04) | | 31/344 (1.74E-02) | | 44/318 (7.89E-04) | |
|  | D<0: | | 11/378 (7.99E-01) | | 10/378 (6.24E-01) | | 7/192 (5.34E-01) | 11/320 (1.52E-01) | | 23/305 (4.49E-02) | | 14/344 (4.22E-01) | | 24/318 (9.88E-03) | |
|  | D>0: | | 21/378 (3.05E-01) | | 22/378 (8.25E-02) | | 10/192 (6.64E-01) | 9/320 (7.27E-01) | | 23/305 (2.04E-02) | | 17/344 (1.40E-02) | | 20/318 (4.20E-02) | |
| Necrotrophic/  Biotrophic | D*: | | 50/608 (3.38E-01) | | 52/608 (1.24E-01) | | 26/318 (5.75E-01) | 34/515 (3.71E-01) | | 43/486 (5.71E-01) | | 41/550 (4.53E-02) | | 52/504 (6.05E-02) | |
|  | D<0: | | 17/608 (7.99E-01) | | 15/608 (6.24E-01) | | 13/318 (5.34E-01) | 16/515 (1.52E-01) | | 19/486 (8.19E-01) | | 18/550 (4.22E-01) | | 25/504 (2.80E-01) | |
|  | D>0: | | 33/608 (3.05E-01) | | 37/608 (5.08E-02) | | 13/318 (7.59E-01) | 18/515 (7.27E-01) | | 24/486 (2.83E-01) | | 23/550 (1.86E-02) | | 27/504 (6.81E-02) | |
| Necrotrophic/  PA | D*: | | 68/950 (6.14E-01) | | 76/950 (1.24E-01) | | 43/482 (5.75E-01) | 47/801 (3.71E-01) | | 71/761 (5.71E-01) | | 65/864 (1.74E-02) | | 93/787 (7.59E-04) | |
|  | D<0: | | 22/950 (8.49E-01) | | 26/950 (6.24E-01) | | 19/482 (5.34E-01) | 23/801 (1.52E-01) | | 34/761 (8.19E-01) | | 29/864 (4.22E-01) | | 49/787 (9.88E-03) | |
|  | D>0: | | 46/950 (3.56E-01) | | 50/950 (8.25E-02) | | 24/482 (6.64E-01) | 24/801 (7.27E-01) | | 37/761 (2.83E-01) | | 36/864 (1.13E-02) | | 44/787 (3.64E-02) | |
| **Total** | **D*:** | | **872/11860** | | **812/11860** | | **476/5706** | **537/9652** | | **715/7949** | | **611/10733** | | **741/8893** | |
| **Total** | **D<0:** | | **331/11860** | | **309/11860** | | **204/5706** | **221/9652** | | **370/7949** | | **329/10733** | | **388/8893** | |
| **Total** | **D>0:** | | **541/11860** | | **503/11860** | | **272/5706** | **316/9652** | | **345/7949** | | **282/10733** | | **353/8893** | |
| **B) Positive Selection** | |  | |  | |  | | |  | |  | |  | |  |
| **Functional Categories** | | **Coding** | | **3' Downstream** | | **3' UTR** | | | **5' Upstream** | | **5'UTR** | | **Introns** | |  |
| CAZymes | | 9/494 (5.7E-01) | | 37/254 (1.4E-01) | | 31/412 (2.5E-01) | | | 30/398 (8.9E-01) | | 22/446 (8.1E-01) | | 21/368 (8.32E-01) | |  |
| Cytochrome P450 | | 4/147 (3.7E-01) | | 8/70 (5.9E-01) | | 9/115 (3.4E-01) | | | 8/82 (8.4E-01) | | 5/121 (8.1E-01) | | 6/128 (8.56E-01) | |  |
| Genus-specific Effectors | | 6/176 (1.6E-01) | | 11/97 (5.9E-01) | | 7/139 (7.8E-01) | | | 11/128 (8.4E-01) | | 7/148 (8.1E-01) | | 3/96 (8.84E-01) | |  |
| Secondary Metabolism | | 21/300 (1.7E-06) | | 23/128 (1.0E-01) | | 25/243 (5.1E-02) | | | 18/169 (8.4E-01) | | 9/269 (8.1E-01) | | 18/232 (2.7E-01) | |  |
| Secreted | | 44/1346 (6.3E-04) | | 97/705 (1.2E-01) | | 83/1130 (1.8E-01) | | | 113/1080 (5.7E-01) | | 56/1225 (8.1E-01) | | 51/1000 (8.56E-01) | |  |
| Secreted Protease | | 3/110 (3.8E-01) | | 11/56 (1.2E-01) | | 11/90 (8.0E-02) | | | 9/85 (8.4E-01) | | 3/101 (8.1E-01) | | 4/98 (8.56E-01) | |  |
| Transcription Factor | | 16/551 (9.6E-02) | | 44/307 (1.4E-01) | | 34/460 (2.5E-01) | | | 33/399 (8.5E-01) | | 20/510 (8.1E-01) | | 37/460 (5.04E-02) | |  |
| Transporter | | 20/662 (5.1E-02) | | 54/313 (1.7E-02) | | 41/555 (2.5E-01) | | | 46/510 (8.4E-01) | | 32/623 (8.1E-01) | | 37/618 (6.39E-01) | |  |
| Virulence Factor | | 39/1445 (3.3E-02) | | 95/754 (2.9E-01) | | 102/1227 (1.9E-02) | | | 100/1127 (8.4E-01) | | 54/1341 (8.1E-01) | | 65/1270 (8.56E-01) | |  |
| **Expresion Categories** | |  | |  | |  | | |  | |  | |  | |  |
| Biotrophic/PA | | 9/379 (2.7E-01) | | 33/191 (4.0E-02) | | 34/320 (4.9E-03) | | | 28/305 (6.0E-01) | | 12/344 (7.9E-01) | | 16/312 (5.69E-01) | |  |
| Necrotrophic/Biotrophic | | 16/615 (1.6E-01) | | 46/317 (7.0E-02) | | 38/514 (1.8E-01) | | | 45/485 (6.0E-01) | | 25/550 (7.9E-01) | | 35/497 (6.43E-02) | |  |
| Necrotrophic/PA | | 26/964 (1.0E-01) | | 69/478 (5.2E-02) | | 70/800 (4.9E-03) | | | 68/760 (6.0E-01) | | 34/864 (7.9E-01) | | 52/768 (6.43E-02) | |  |
| **Total** | | **224/11995** | | **668/5693** | | **613/9648** | | | **728/7944** | | **456/10724** | | **457/8742** | |  |
| Values indicate number of sequences in the outlier (or PS) set / total number of sequences analyzed at each category. Values between brackets show the p-value from the Fisher’s exact test after correction for multiple comparisons (FDR<0.05, in red significant p-values). Tajima’s D outliers columns indicate the kind of outlier considered: D* (D<5th percentile or D>95th percentile), D*<0 (D<5th percentile), D*>0 (D > 95th percentile). | | | | | | | | | | | | | | |  |

| **Table S4.** **Interesting genes under positive selection in the coding sequence.** | | | | | | | |
| --- | --- | --- | --- | --- | --- | --- | --- |
| **Gene ID** | **Annotation** | **Expression in Cg** | **Secreted** | **PhiBase ID** | **Phenotype of mutant** | **Pathogen species** | **Experimental host** |
| GLRG_00054 | ABC transporter | S/S/S | NO | PHI:202 | Reduced virulence | *Botrytis cinerea* | Grape |
| GLRG_00247 | ABC-2 type transporter | D/D/S | NO | PHI:258 | Reduced virulence | *Gibberella pulicaris* | Potato |
| GLRG_00264 | Hypothetical protein | S/D/S | YES | - | - | *-* | - |
| GLRG_00468 | ABC transporter | S/S/S | NO | PHI:1018 | Loss of pathogenicity | *Magnaporthe oryzae* | Rice |
| GLRG_00469 | AMP-binding enzyme | S/S/S | NO | PHI:160 | Loss of pathogenicity | *Alternaria alternata* | Apple |
| GLRG_00513 | ABC-2 type transporter | S/S/S | NO | PHI:258 | Reduced virulence | *Gibberella pulicaris* | Potato |
| GLRG_00514 | ABC transporter | S/S/S | NO | PHI:267 | Reduced virulence | *Candida albicans* | Mouse |
| GLRG_00920 | Amino acid adenylation | S/S/S | NO | PHI:12 | Loss of pathogenicity | *Cochliobolus carbonum* | Maize |
| GLRG_01586 | Hypothetical protein | S/U/S | YES | - | - | *-* | - |
| GLRG_01804 | Hypothetical protein | U/U/S | YES | - | - | *-* | - |
| GLRG_01845 | Transcription factor | S/S/S | NO | PHI:169 | Loss of pathogenicity | *Colletotrichum lindemuthianum* | Bean |
| GLRG_01860 | Beta-ketoacyl synthase | S/S/S | NO | PHI:55 | Reduced virulence | *Cochliobolus heterostrophus* | Maize |
| GLRG_02650 | Adhesin protein Mad1 | S/S/S | YES | - | - | *-* | - |
| GLRG_02963 | Hypothetical protein | S/S/S | NO | PHI:404 | Reduced virulence | *Magnaporthe oryzae* | Rice |
| GLRG_03150 | Hypothetical protein | S/S/S | NO | PHI:211 | Reduced virulence | *Candida albicans* | Mouse |
| GLRG_03507 | Beta-ketoacyl synthase | S/S/S | NO | PHI:101 | Reduced virulence | *Aspergillus fumigatus* | Mouse |
| GLRG_03511 | Beta-ketoacyl synthase | S/S/S | NO | PHI:55 | Reduced virulence | *Cochliobolus heterostrophus* | maize |
| GLRG_04079 | Hypothetical protein | S/D/D | YES | - | - | *-* | - |
| GLRG_04142 | Hypothetical protein | U/U/S | YES | - | - | *-* | - |
| GLRG_05009 | Hypothetical protein | S/S/S | YES | - | - | *-* | - |
| GLRG_05053 | Hypothetical protein | S/S/S | NO | PHI:864 | Loss of pathogenicity | *Aspergillus fumigatus* | Mouse |
| GLRG_05268 | Methyltransferase | U/U/S | NO | PHI:482 | Reduced virulence | *Aspergillus fumigatus* | Mouse |
| GLRG_05919 | Hypothetical protein | S/S/S | YES | - | - | *-* | - |
| GLRG_05958 | Glycosyl hydrolase | S/S/S | YES | PHI:1071 | Loss of pathogenicity | *Ustilago maydis* | Maize |
| GLRG_06092 | Mannosyltransferase CMT1 | S/D/D | NO | PHI:455 | Loss of pathogenicity | *Cryptococcus neoformans* | Mouse |
| GLRG_06331 | WD domain | S/S/S | NO | PHI:211 | Reduced virulence | *Candida albicans* | Mouse |
| GLRG_06371 | Hypothetical protein | S/S/S | YES | - | - | *-* | - |
| GLRG_06485 | Hypothetical protein | S/D/S | YES | - | - | *-* | - |
| GLRG_06732 | Cytochrome P450 | S/S/S | NO | PHI:438 | Reduced virulence | *Botrytis cinerea* | Bean |
| GLRG_06861 | Hypothetical protein | S/S/S | YES | - | - | *-* | - |
| GLRG_07140 | Hypothetical protein | D/D/S | YES | - | - | *-* | - |
| GLRG_07145 | Cytochrome P450 | S/S/S | NO | PHI:438 | Reduced virulence | *Botrytis cinerea* | Bean |
| GLRG_07254 | Alpha-L-rhamnosidase | S/S/S | YES | - | - | *-* | - |
| GLRG_07434 | Beta-ketoacyl synthase | S/U/U | NO | PHI:325 | Effector (plant avirulence determinant) | *Magnaporthe oryzae* | Rice |
| GLRG_07527 | Hypothetical protein | S/S/S | YES | - | - | *-* | - |
| GLRG_07678 | Hypothetical protein | S/S/S | YES | - | - | *-* | - |
| GLRG_07748 | Hypothetical protein | S/D/S | YES | - | - | *-* | - |
| GLRG_07825 | Sugar lactone oxidase | S/S/S | NO | PHI:197 | Reduced virulence | *Candida albicans* | Mouse |
| GLRG_08161 | Hypothetical protein | S/S/S | YES | - | - | *-* | - |
| GLRG_08505 | Hypothetical protein | S/S/S | YES | - | - | *-* | - |
| GLRG_08566 | Hypothetical protein | S/S/S | YES | - | - | *-* | - |
| GLRG_08615 | MFS Transporter | S/S/S | NO | PHI:141 | Reduced virulence | *Cercospora kikuchii* | Soybean |
| GLRG_08620 | Beta-ketoacyl synthase | S/S/S | NO | PHI:433 | Reduced virulence | *Cercospora nicotianae* | Tobacco |
| GLRG_08878 | Hypothetical protein | S/S/S | YES | - | - | *-* | - |
| GLRG_08901 | Hypothetical protein | S/S/S | YES | - | - | *-* | - |
| GLRG_09110 | Hypothetical protein | S/D/D | YES | PHI:256 | Reduced virulence | *Magnaporthe oryzae* | Rice |
| GLRG_09221 | DEAD/DEAH box helicase | S/S/S | NO | PHI:423 | Loss of pathogenicity | *Claviceps purpurea* | Ergot |
| GLRG_09382 | Tannase/feruloyl esterase | S/S/S | YES | - | - | *-* | - |
| GLRG_09394 | Hypothetical protein | U/U/S | YES | - | - | *-* | - |
| GLRG_09842 | AMP-binding | S/S/S | NO | PHI:325 | Effector (plant avirulence determinant) | *Magnaporthe oryzae* | Rice |
| GLRG_10257 | MFS Transporter | S/S/S | NO | PHI:511 | Reduced virulence | *Candida albicans* | Mouse |
| GLRG_10317 | Beta-ketoacyl synthase | S/S/S | NO | PHI:55 | Reduced virulence | *Cochliobolus heterostrophus* | maize |
| GLRG_10367 | AMP-binding | S/S/S | NO | PHI:325 | Effector (plant avirulence determinant) | *Magnaporthe oryzae* | Rice |
| GLRG_10457 | Hypothetical protein | S/D/D | YES | - | - | *-* | - |
| GLRG_11626 | Beta-ketoacyl synthase | S/S/S | NO | PHI:325 | Effector (plant avirulence determinant) | *Magnaporthe oryzae* | Rice |
| GLRG_11821 | Linoleate diol synthase | D/D/S | NO | PHI:496 | Increased virulence (Hypervirulence) | *Aspergillus fumigatus* | Mouse |
| GLRG_11938 | Hypothetical protein | S/D/D | YES | - | - | *-* | - |
| Gene Id and Annotation according to the Broad Institute annotation. Expression in *Cg* indicate whether the gene is UP (U) regulated DOWN (D) regulated or STABLE (S) at each time point during infection on maize (Biotrophic/PA / Necrotrophic/PA / Necrotrophic/Biotrophic) according to (O’Connell et al. 2012). PhiBase ID: Best match on PhiBase (Winnenburg et al. 2006). | | | | | | | |

# 4. Supplementary Figures

Fig. S1. Sliding-windows analysis of Tajima’s D values across the genome. **A)** Tajima’s D values for 5kb windows with 500bp slides (jumps), comprising 98,624 windows covering the 13 chromosomes plus unanchored supercontigs. Dashed lines represent 5^th^ and 95^th^ percentiles. **B)** Percentage of coding sequence (CDS) and **C)** Percentage of repetitive DNA at each window. Similar results were obtained using non-overlapping windows (5000bp slides, comprising 9,862 windows).


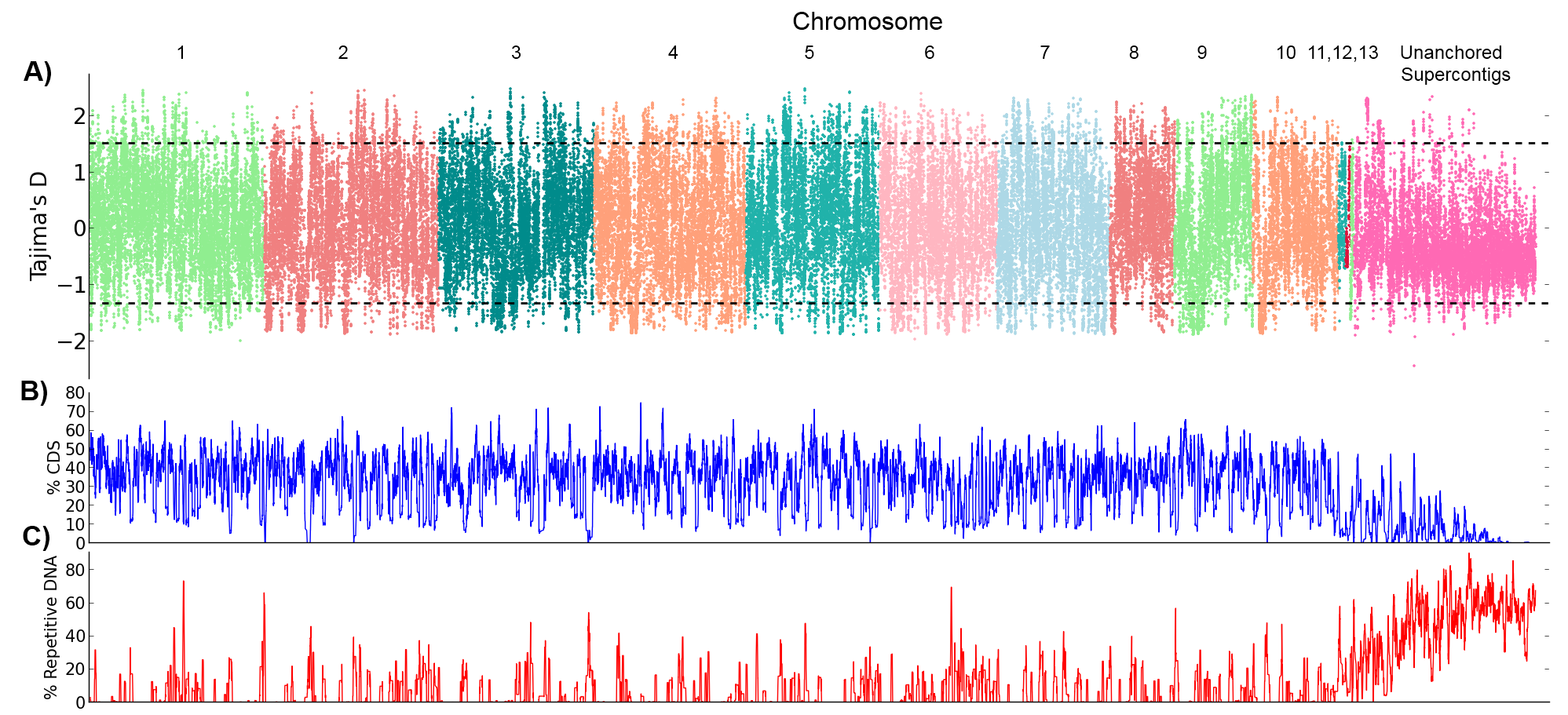


**Fig. S2. Dotplots showing the relationship between the pairwise nucleotide differences per site (Π) and the percentage of coding sequence in the window. A)** Values obtained using non overlapping 5kb windows. **B)** Values obtained using 5kb windows with 500bp slides (jumps). In both cases Spearman correlation value: ρ = -0.49 (p<0.0001) and Pearson correlation value: r = -0.53 (p<0.0001).


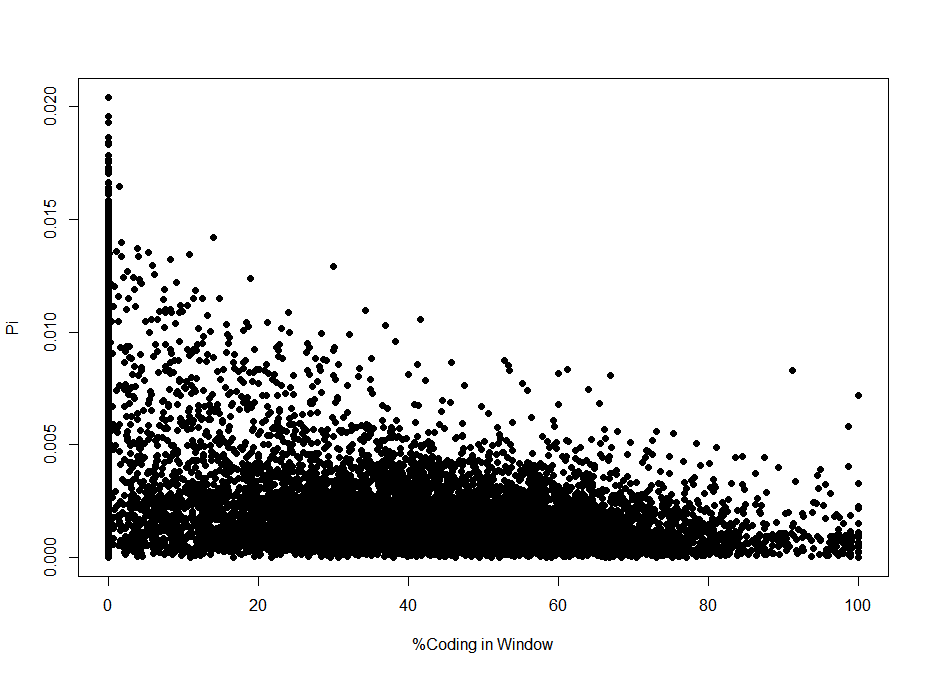


**A)**


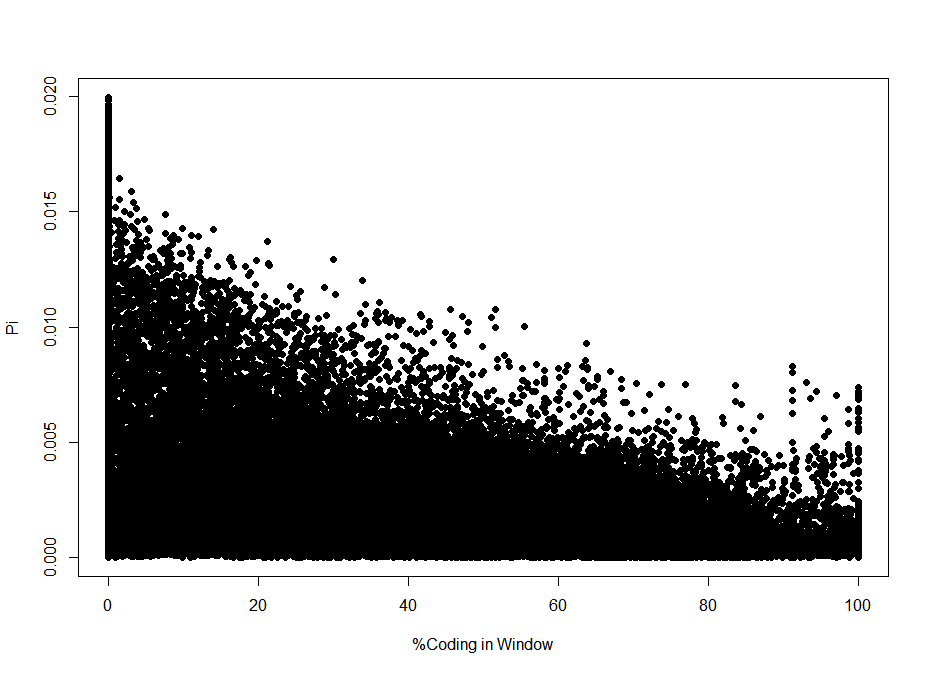


**B)**

## Fig. S3. Venn diagram showing gene intersections between different classes of non-coding sequences under positive selection.

**
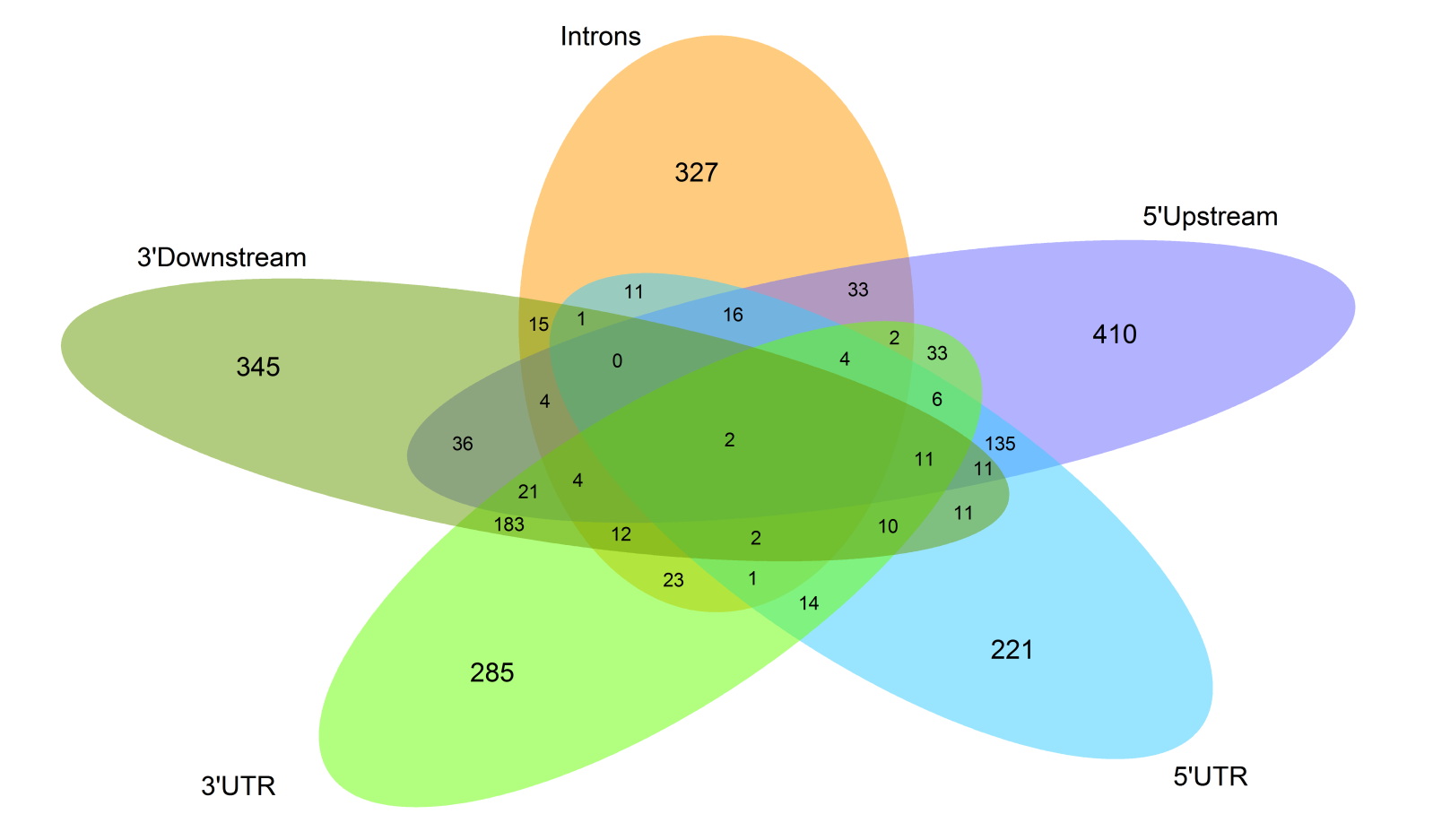
**

# 5. References

Achaz G. 2008. Testing for Neutrality in Samples With Sequencing Errors. Genetics. 179:1409–1424. doi: 10.1534/genetics.107.082198.

Baek J-M, Kenerley CM. 1998. The arg2 Gene of Trichoderma virens: Cloning and Development of a Homologous Transformation System. Fungal Genet. Biol. 23:34–44. doi: 10.1006/fgbi.1997.1025.

Bickel RD, Dunham JP, Brisson JA. 2013. Widespread Selection Across Coding and Noncoding DNA in the Pea Aphid Genome. G3 GenesGenomesGenetics. 3:993–1001. doi: 10.1534/g3.113.005793.

Biswas S, Akey J. 2006. Genomic insights into positive selection. Trends Genet. 22:437–446. doi: 10.1016/j.tig.2006.06.005.

Carlson CS et al. 2005. Genomic regions exhibiting positive selection identified from dense genotype data. Genome Res. 15:1553–1565. doi: 10.1101/gr.4326505.

Gaffney DJ, Keightley PD. 2006. Genomic Selective Constraints in Murid Noncoding DNA. PLoS Genet. 2:e204. doi: 10.1371/journal.pgen.0020204.

Haygood R, Fedrigo O, Hanson B, Yokoyama K-D, Wray GA. 2007. Promoter regions of many neural- and nutrition-related genes have experienced positive selection during human evolution. Nat. Genet. 39:1140–1144. doi: 10.1038/ng2104.

Hutter S, Vilella AJ, Rozas J. 2006. Genome-wide DNA polymorphism analyses using VariScan. BMC Bioinformatics. 7:409. doi: 10.1186/1471-2105-7-409.

Kelley JL, Madeoy J, Calhoun JC, Swanson W, Akey JM. 2006. Genomic signatures of positive selection in humans and the limits of outlier approaches. Genome Res. 16:980–989. doi: 10.1101/gr.5157306.

Li H, Ruan J, Durbin R. 2008. Mapping short DNA sequencing reads and calling variants using mapping quality scores. Genome Res. 18:1851–1858. doi: 10.1101/gr.078212.108.

O’Connell RJ et al. 2012. Lifestyle transitions in plant pathogenic Colletotrichum fungi deciphered by genome and transcriptome analyses. Nat. Genet. 44:1060–1065. doi: 10.1038/ng.2372.

Przeworski M, Hudson RR, Di Rienzo A. 2000. Adjusting the focus on human variation. Trends Genet. 16:296–302. doi: 10.1016/S0168-9525(00)02030-8.

Vaillancourt LJ, Hanau RM. 1992. Genetic and morphological comparisons of Glomerella (Colletotrichum) isolates from maize and from sorghum. Exp. Mycol. 16:219–229. doi: 10.1016/0147-5975(92)90030-U.

Winnenburg R et al. 2006. PHI-base: a new database for pathogen host interactions. Nucleic Acids Res. 34:D459–D464. doi: 10.1093/nar/gkj047.

Wong WSW, Nielsen R. 2004. Detecting Selection in Noncoding Regions of Nucleotide Sequences. Genetics. 167:949–958. doi: 10.1534/genetics.102.010959.
